# Employing AI tools to predict features for dental care use in the United States during the global respiratory illness outbreak

**DOI:** 10.3389/fpubh.2025.1692540

**Published:** 2026-01-13

**Authors:** Preeti Pushpalata Zanwar, Hamid-Reza Kohan-Ghadr, Vijayakumar Thirumalai, Suad Ghaddar, Shuo Jim Huang, Beth Harkness, Eben Rey, Romanch Shah, Sairaja Reddy Kurelli, Jay S. Patel, Luca Calzoni, Elif Dede Yildirim, Deborah Guadalupe Duran

**Affiliations:** 1 Center for Health and Social Sciences, University of Chicago, Chicago, IL, United States; 2 Texas A&M University Health, Irma Lerma Rangel College of Pharmacy, College Station/Kingsville, TX, United States; 3 SysBioSolutions LLC, Portage, MI, United States; 4 HealthPartners Institute, Minneapolis, MN, United States; 5 University of Texas Rio Grande Valley, Edinburg, TX, United States; 6 Institute for Health Computing, University of Maryland, North Bethesda, MD, United States; 7 Inova Health System, Fairfax, VA, United States; 8 NEIO Systems LLC, Los Angeles, CA, United States; 9 Schulich School of Medicine and Dentistry, Western University, London, ON, Canada; 10 University of Texas Austin, Sugar Land, TX, United States; 11 Temple University Kornberg School of Dentistry, Philadelphia, PA, United States; 12 University of Pittsburgh, Pittsburgh, PA, United States; 13 National Institutes on Minority Health and Health Disparities, Bethesda, MD, United States

**Keywords:** artificial intelligence, cloud computing, dental population health, dental public health, dental health services research, dental health policy, cross-sector collaboration (partnerships)

## 1 Introduction

Oral health is strongly associated with overall health. Prior research has demonstrated the benefits of regular dental visits to prevent initiation and progression of systemic diseases such as diabetes and cardiovascular diseases (1, 2). Prior research has also demonstrated the impacts of dental treatments such as scaling, and root planning on reductions in chronic inflammation which can further help in managing chronic conditions like diabetes (3). The American Dental Association recommends routine visits to a dentist once or twice a year for children, adults and older adults to manage overall health (4, 5). Some individuals may need to visit a dentist more than twice a year to maintain good oral health and overall health (4). Despite these recommendations, access to dental care in the United States (U.S.) has long been a critical public health issue, with only half of the U.S. population over the age of two having ever accessed dental care (6) and 72 million or approximately 27% of adults not having dental insurance (7). Moreover, there is socioeconomic gradient in access to oral health in the U.S. (8). Increasing levels of income and education are associated with higher frequency of annual dental care visits on a population level. That is, those with higher income and higher education are more likely to have dental insurance which is a key factor for access to routine and timely dentist visits. Additionally, even among those with higher socio-economic status, gaps in oral health and dental care access continue to exist, as these challenges are influences by broader structural issues within the health care system and by factors such as transportation and housing conditions (9, 10).

Factors related to low-income status, race, ethnicity, and rural status have further contributed to the lack of, and variability of dental care access thereby contributing to poor oral health outcomes (11, 12). Challenges in accessing dental public health services have been associated with the exacerbation of systemic health ailments, including multimorbidity, diabetes, heart and lung disease, dementia, arthritis and complications during pregnancy among ethnically and culturally diverse populations (12–14). As such, these pronounced disparities in access to, quality of, and affordability of routine primary oral healthcare have impacted a wide range of communities, particularly those with disabilities, dual-eligible, and racial and/or ethnic minority populations who face a greater burden of health disparities (15–18). In a recent study, those with a low-income status and less than high school education reported higher unmet dental needs than their more affluent and educated counterparts (18). When comparing population groups, Black/African and Hispanic/Latino Americans have reported higher levels of untreated dental disease compared to White Americans (18). In the context of the recent pandemic of 2020–2022, these disparities were further compounded due to urgent oral treatments taking precedence over routine visits (19). This shift resulted in a notable decrease in oral healthcare utilization in the U.S. by 18 million users in the first year of the pandemic, with lasting repercussions and a gradual, slow recovery to pre-pandemic levels (19). While research has been done on what factors and obstacles in healthcare were associated with barriers to dental visits before 2020 (15–18), much less has been examined regarding the factors associated with decline in dental care utilization beyond age-related survey stratifications of dental visits during the peak of the global public health crisis in 2021 (19). A study examined dental utilization and oral conditions from 2019 to 2020 using Electronic Health Records (EHRs) from federally qualified health centers in the U.S. and found visits to dental care providers decreased more in comparison to other health providers during the shelter-in-place orders during the 2020–2021 (20). Another study found 47% of respondents delayed visiting a dentist for dental check-ups, pain, and for seeking care for planned treatment during the recent pandemic of 2020–2022 (21).

Although these studies provided meaningful insights into oral health disparities and access to care issues, the barriers can be further studied by utilizing comprehensive survey datasets and using AI models that would help identify and uncover complex, non-linear relationships between confounding factors that traditional statistical methods may overlook. The statistical methods have been widely used in prior research and limited research has employed Al models on U.S. federal nationally representative survey datasets, a gap we identified. Therefore, to address this gap, our study examines dental care utilization during the second year of the pandemic by applications of multiple AI/ML models to uncover complex, interacting social and health determinants that are not easily captured through traditional statistical approaches.

Our aims for this data report were to examine the major contributors that drive oral healthcare visits and identify barriers that have limited access during the pandemic using the nationally representative Medical Expenditure Panel Survey (MEPS). Specifically, the objectives were to (1) demonstrate how Al approaches can be used with nationally representative survey data; (2) provide preliminary findings from multiple Al models regarding top factors associated with dental services utilization in the U.S. using feature importance; and (3) identify to which extent these factors vary across different Al models. To achieve these objectives, multiple AI models were utilized to process and analyze the MEPS dataset. By leveraging AI tools, preliminary findings were identified regarding the top factors for dental visits in the U.S. along with the extent to which these factors vary across AI models.

## 2 Data sources and research methodology

In this data report, we describe the dataset, outline the analytic workflow, and present key model-derived findings rather than developing or testing any causal hypotheses.

### 2.1 Study design

We used the cross-sectional Medical Expenditure Panel Survey data spanning year 2021.

### 2.2 Dataset description

The data report utilizes the nationally representative MEPS, the most comprehensive data collected from American households on healthcare use, costs (including out-of-pockets from self/family, healthcare expenditures and payments to public and private payers), and health insurance coverage in the U.S. MEPS includes information on dental insurance, including on health and dental service use for the American households. Importantly, in recent years MEPS has been oversampling certain population groups and collecting demographic and social factors and satisfaction with care. MEPS has been oversampling Hispanics, Blacks, and Asians, as well as low-income households, to improve the precision and reliability of estimates for these groups. This is because these groups are underrepresented in a standard national sample, and oversampling allows researchers to have a statistically significant sample size for detailed analysis of their healthcare utilization and expenditures. The primary sponsor for MEPS is the Agency of Healthcare Research & Quality (AHRQ); MEPS is co-sponsored by the National Center for Health Statistics. MEPS is a sub-sample of the National Health Interview Survey (NHIS), and MEPS data collection has been ongoing annually since 1996. More information on MEPS is available from https://meps.ahrq.gov/mepsweb/ (22). MEPS data files, documentation, and codebook are available to download from the MEPS website where programming statements in statistical, coding, and visualization packages in Stata, SAS, SPSS, and R are provided. Additionally, reference guides from MEPS workshops on how to work with MEPS data are made available by AHRQ at GitHub from https://github.com/HHS-AHRQ/MEPS/blob/master/Quick_Reference_Guides/README.md (23).

### 2.3 Data collection period

For this data report, the MEPS full-year consolidated household file, HC-233, from the 2021 calendar year (the second year of the pandemic) was analyzed. Person-level de-identified full-year household consolidated file MEPS data on healthcare utilization was used, with a focus on predictive factors. These factors include sociodemographic factors such as age, gender, race/ethnicity, socioeconomic factors such as education, household income, and enabling factors such as dental insurance, health behaviors, such as smoking status for dental visits during 2021.

### 2.4 Conceptual framework

The National Institute on Minority Health and Health Disparities (NIMHD) and the National Institute on Aging Health Disparities Research Framework across domains and levels of influence was utilized to conceptualize, operationalize and provide structure to our methodological approach (24, 25). While MEPS does not collect data on all elements represented within the NIMHD framework (e.g., biological and physical environmental domain), a wide range of factors (e.g., within health behaviors, sociocultural environmental, healthcare system) and levels (e.g., individual, interpersonal, family, community and societal/population) that impact health outcomes are part of MEPS routine data collection. For our data report, individual level factors and healthcare system domains that can influence oral health outcomes for a person and for community-living non-institutionalized civilian U.S. population were examined. Dental utilization was conceptualized as a proxy for health seeking behavior of an individual based on the Anderson Health Behavior Model for health service utilization (26, 27).

### 2.5 Description of research team

Our multidisciplinary and cross-sectorial team from academia, the private sector, and government, is comprised of a dentist, big data scientists, a nursing professional, and researchers focused on healthcare delivery, health services research and outcomes, health policy, and health disparities, including data scientists from the NIMHD. The team is one of the invited and facilitated teams of the Science Collaborative for Health and Artificial Intelligence Reduction of Errors (SCHARE) led by NIMHD and the National Institute of Nursing Research (NINR) which aims to upskill novice untrained users in data science and to advance use of transparency and sophisticated inquiry, and to advance and foster paradigm shifts in population health, SDOH, and health disparities research. More information on SCHARE is available at https://nimhd.nih.gov/resources/schare/ (28). SCHARE provides skills-based theoretical training via (1) hands-on teaching sessions (Think-a-thons), tutorials, resources, (e.g. for Python coding, using the cloud-computing tools, statistics, Al based approaches and error reduction tools, and ethical research) and (2) project-based applied training in data science via cross-disciplinary and cross-level mentoring to facilitate collaborative research projects on population health research, including chronic diseases, health disparities and health outcomes. Our team on SCHARE was facilitated by NIMHD beginning April 2024.

### 2.6 Online platform and data repository

The SCHARE ecosystem is comprised of (1) a Google cloud-based and secured Terra collaborative workspace with Jupyter notebooks with human readable executable documents and Python programming codes and libraries that can be readily employed for large-scale advanced data analysis, and (2) a compilation of SCHARE and Google hosted public datasets and SCHARE hosted project datasets on SDOH, access and quality of care, health outcomes, and clinical data and EHRs. MEPS is one of the public datasets hosted by SCHARE.

### 2.7 Al approach

In this data report, the MEPS 2021 full-year file, consisting of 28,226 person/family level observations and 1,488 unique variables, was described and analyzed. Data processing steps are detailed for handling missing values, normalizing continuous variables (e.g., dental visits) and encoding categorical variables. Additionally, guidance is provided on interpreting the MEPS dataset to understand variations in dental care access, reusing the data for improving health services applicable to all individuals, and conducting research elucidating and comparing important factors for dental care access among U.S. populations historically experiencing significant challenges.

#### 2.7.1 Al Classification models

Four robust Al approaches and models were chosen, namely Decision Tree Regressor, Random Forest Regressor, XGBoost Regressor, and LightGBM Regressor for their efficiency in handling structured, large- scale health survey data like MEPS. Random Forest and Decision Tree offer interpretability and robustness against overfitting, making them well-suited for analyzing dental visit patterns (25). XGBoost and LightGBM are known for their scalability and predictive accuracy, particularly in identifying key features from high-dimensional data (29–31). These models collectively allow for a thorough examination of the factors affecting dental visit utilization in the U.S. The target variable for all models was dental visits, with a total of 1,488 variables included in the study. To focus on the factors that most influence how often people visit the dentist, certain variables that could introduce unnecessary complexity or bias into the models were excluded. Specifically, while the total amount paid by families for dental care variable was retained in the models, variables that break down payments by specific sources excluded, including total charges, out-of-pocket payments, and payments from Medicare, Medicaid, and private insurance. Additionally, survey-specific identifiers, variance estimation parameters, demographic identifiers, and statistical weights used for producing nationally representative estimates were also excluded (see Supplementary Table 1 for details). This decision was made to streamline the analysis and maintain a clear focus on the most critical variables impacting dental visit frequency, without being confounded by peripheral or redundant data,

#### 2.7.2 Two-step Al model building

Two primary objectives were followed to address the research goals. For the first objective, identification of significant features that predict whether an individual had no dental visits vs. at least one dental visit was aimed, given the data's imbalance, with a higher prevalence of zero dental visits. To achieve this, binary classification models were performed to distinguish between those with zero dental visits and those with at least one, allowing for the identification of barriers and facilitators associated with dental care utilization and the characteristics of non-utilizers. An 80/20 stratified train–test split was also used to maintain the class distribution between individuals with and without dental visits across training and testing subsets. For the second objective, analysis focused on individuals with 1–2 dental visits, which represent routine preventive care, vs. those with more than two visits, potentially indicative of therapeutic visits. A binary classification model was employed to differentiate between these two groups, aiming to identify the factors contributing to more frequent dental visits.

#### 2.7.3 Evaluation metrics

In this study, the natural class distribution was retained to reflect real-world utilization patterns, and model performance was evaluated using metrics (e.g., F1 and ROC-AUC) that are appropriate for imbalanced data. Model performance metrics (accuracy, precision, recall, F1 score, and ROC-AUC) and feature importance rankings served as the statistical basis for interpreting key predictors of dental utilization (29–31). These metrics provide a comprehensive view of the models' performance, capturing both their predictive accuracy and their ability to balance false positives and false negatives. Feature importance values were calculated after model training to summarize each model's internal weighting of predictors and were used for interpretability only; model performance was evaluated independently based on the accuracy-related metrics above. The model with the best combination of performance metrics, as assessed via minimized connectivity and maximized Dunn Index, was selected as the optimal model for predicting dental services utilization. Model selection was based on either overall dominance across all metrics, or superior performance on metrics most relevant to the outcome data structure. The top 10 features were identified based on their ranking derived from the feature Importance scores.

## 3 Results

Objective 1 aimed to identify the significant factors associated with no dental visits vs. at least one dental visit. Four binary classification models (i.e., Decision Tree, Random Forest, XGBoost, and LightGBM) were developed to distinguish between individuals who did not utilize dental care and those who had at least one visit. Table 1 presents performance metrics, including accuracy, precision, recall, F1 score, and ROC-AUC, alongside the top ten features ranked by feature importance for each model, providing insights into the key determinants of dental visit behavior. Performance metrics and top ten predictive features for each model are summarized in Table 1. Based on all performance indicators, overall XGBoost's performance was superior followed by LigthtGBM. XGBoost provided the highest values for overall model metrics, i.e., accuracy (0.864), precision (0.828), recall (0.843), F1 score (0.835), and ROC-AUC (0.939) (Table 1).

**Table 1:** Top ten predictive features for no dental visits vs. at least one dental visit across four artificial intelligence models and their performance, Medical Expenditure Panel Survey, year 2021.

**Model**	**Performance indicators**	**Top ten features**			
		**Rank**	**Feature**	**Importance**	**Description**
Decision tree	Accuracy 0.74 Precision 0.68 Recall 0.68 F1 Score 0.68 ROC AUC 0.73	1	TOTTCH21	0.193	Total Healthcare Charges for 2021 excluding Prescription Medicines
		2	TOTSLF21	0.055	Total Amount Paid by Self/Family in 2021
		3	HIDEG	0.047	Highest Degree When First Entered MEPS
		4	OBVTCH21	0.035	Office-Based Provider Visit Charges for 2021
		5	TOTEXP21	0.021	Total Healthcare Expenditures for 2021
		6	AGE53X	0.021	Age Round5/3 (Edited/Imputed)
		7	OBVEXP21	0.014	Total Office-Based Expenditures for 2021
		8	RXSLF21	0.010	Total Amount Paid for Prescription Medicines by Self/Family
		9	LSTETH53	0.010	Lost All Upper and Lower Teeth among age >17 years, Round 5/3
		10	VISTCH21	0.010	Glasses/Contact Lenses Charges for 2021
Random forest	Accuracy 0.79 Precision 0.74 Recall 0.73 F1 Score 0.84 ROC AUC 0.94	1	TOTTCH21	0.033	Total Healthcare Charges for 2021 excluding Prescription Medicines
		2	TOTEXP21	0.025	Total Healthcare Expenditures for 2021
		3	TOTSLF21	0.024	Total Amount Paid by Self/Family in 2021
		4	TOTPTR21	0.021	Total Amount Paid by Private and Tri Care
		5	TOTPRV21	0.017	Total Amount Paid by Private Insurance 2021
		6	OBDEXP21	0.012	Total Office-Based Doctor Expenditures for 2021
		7	OBVTCH21	0.011	Office Based Provider Visit Charges for 2021
		8	POVLEV21	0.011	Family Income as % of Poverty Line - Continuous
		9	OBVEXP21	0.011	Total Office-Based Expenditures for 2021
		10	OBDTCH21	0.009	Office-Based Physician Visit Charges for 2021
XGBoost	Accuracy 0.86 Precision 0.83 Recall 0.84 F1 Score 0.84 ROC AUC 0.94	1	CHLIMI42	0.023	CSHCN: Limited in Anyway (age 0-17 years) R4/2
		2	HIDEG	0.020	Highest Degree When First Entered MEPS
		3	TOTTCH21	0.017	Total Healthcare Charges for 2021 excluding Prescription Medicines
		4	ADHDADDX	0.014	ADHD/ADD Diagnosis (age 5-17 years)
		5	SDUNEXPEXP	0.012	SDOH: Cover Unexpected Expense
		6	AGE53X	0.011	Age Round5/3 (Edited/Imputed)
		7	LSTETH53	0.011	Lost All Upper and Lower Teeth among age >17 years, Round 5/3
		8	NERVAF42	0.011	Problem Feeling Nervous/Afraid (age 5-17 years) -R4/2
		9	INSMA21X	0.011	Covered by Hospital/Medical Insurance in March 2021
		10	RTHLTH31	0.010	Perceived Health Status RD 3/1
LightGBM	Accuracy 0.86 Precision 0.82 Recall 0.84 F1 Score 0.83 ROC AUC 0.94	1	TOTTCH21	223.0	Total Healthcare Charges for 2021 excluding Prescription Medicines
		2	TOTSLF21	183.0	Total Amount Paid by Self/Family in 2021
		3	OBVTCH21	140.0	Office-Based Provider Visit Charges for 2021
		4	OBVSLF21	89.0	All Office Visits Amount Paid for by Self/Family in 2021
		5	TOTPRV21	80.0	Total Amount Paid by Private Insurance in 2021
		6	RXSLF21	78.0	Total Amount Paid for by Prescription Meds by Self/Family in 2021
		7	TOTEXP21	73.0	Total Healthcare Expenditures for 2021
		8	VISSLF21	59.0	Glasses/Contact Lenses Self/Family Amount for 2021
		9	OBVEXP21	50.0	Total Office Based Expenditures in 2021
		10	OTHSLF21	48.0	Other Equipment/Supplies Amount for Self/Family in 2021

MEPS, Medical Expenditure Panel Survey; ROC-AUA, Receiver Operating Characteristic Area Under the Curve; F1, mean score of precision and recall, a score of 1 indicates good performance while low score indicates poor model performance. ROC-AUA, Receiver Operating Characteristic Area Under the Curve; F1, mean score of precision and recall, a score of 1 indicates good performance while low score indicates poor model performance.

Objective 2 aimed to differentiate between individuals with one or two dental visits (representing routine preventive dental care) and those with more than two visits (potentially indicative of therapeutic care). Binary classification models were applied to identify the factors associated with more frequent dental visits. Table 2 highlights the performance metrics (accuracy, precision, recall, F1 score, and ROC-AUC) for Decision Tree, Random Forest, XGBoost, and LightGBM models and top ten features ranked by their importance for predicting dental visit frequency. While XGBoost has slightly better recall and F1 score, LightGBM provided slightly higher precision and ROC-AUC. Given that ROC-AUC is a key metric for classification tasks with imbalanced data, LightGBM performed superior to XGBoost as the better model in this particular case, due to its higher ROC-AUC of 0.782 compared to 0.769 for XGBoost.

**Table 2:** Model performance and key features for predicting dental visit frequency for individuals with 1-2 dental visits representing routine preventive care and those with more than two visits potentially indicative of therapeutic care during the global respiratory illness outbreak, Medical Expenditure Panel Survey, year 2021.

**Model**	**Performance indicators**	**Top ten features**			
		**Rank**	**Feature**	**Importance**	**Description**
Decision tree	Accuracy 0.68 Precision 0.43 Recall 0.43 F1 Score 0.43 ROC AUC 0.60	1	TOTSLF21	0.112	Total Amount Paid by Self/Family in 2021
		2	TOTEXP21	0.030	Total Healthcare Expenditures in 2021
		3	OBVSLF21	0.026	Amount Paid for All Office Visits by Self/Family in 2021
		4	RXSLF21	0.017	Total Amount Paid for Prescription Medicines by Self/Family
		5	TOTTCH21	0.014	Total Healthcare Charges for 2021 excluding Prescription Medicines
		6	OBVEXP21	0.013	Total Office Based Expenditures in 2021
		7	OTHSLF21	0.013	Amount Paid for Other Equipment/Supplies by Self/Family in 2021
		8	VISSLF21	0.012	Amount Paid for Glasses/Contact Lenses by Self/Family in 2021
		9	OPTSLF21	0.011	All Amount Paid for Outpatient Dept Visits (Facility + Doctor) by Self/Family in 2021
		10	POVLEV21	0.01	Income As % of Poverty Line-Continuous
Random forest	Accuracy 0.73 Precision 0.66 Recall 0.11 F1 Score 0.19 ROC AUC 0.71	1	TOTSLF21	0.025	Total Amount Paid by Self/Family in 2021
		2	TOTTCH21	0.022	Total Healthcare Charges for 2021 excluding Prescription Medicines
		3	TOTEXP21	0.02	Total Healthcare Expenditures for 2021
		4	TOTPRV21	0.017	Total Amount Paid by Private Insurance in 2021
		5	TOTPTR21	0.011	Total Amount Paid by Private and Tricare in 2021
		6	OBVEXP21	0.009	Total Office Based Expenditures in 2021
		7	OBVTCH21	0.008	Office-Based Provider Visit Charges for 2021
		8	POVLEV21	0.007	Family Income as % of Poverty Line Continuous
		9	OBDEXP21	0.007	Total Office Based Doctor Expenditures in 2021
		10	AGE21X	0.007	Age as of 12/31/2021 (Edited/Imputed)
XGBoost	Accuracy 0.77 Precision 0.64 Recall 0.40 F1 Score 0.50 ROC AUC 0.77	1	ACTDTY42	0.009	Military Full-time Active Duty - Round 4/2
		2	AGE21X	0.007	Age as of 12/31/2021 (Edited/Imputed)
		3	ERTOT21	0.007	Number of Emergency Room Visits in 2021
		4	TOTSLF21	0.007	Total Amount Paid by Self/Family in 2021
		5	ACTLIM31	0.007	Any Limitation in Work/Housework/School - Round 3/1
		6	SDPAYBASICS	0.006	Social Determinants of Health: How Hard Pay Basics?
		7	ERTSLF21	0.006	Amount Paid by Self/Family For Emergency Room (Facility + Doctor) in 2021
		8	TOTEXP21	0.005	Total Healthcare Expenses 2021
		9	IPFSLF21	0.005	Amount Paid by Self/Family For Inpatient Hospital Stays (Facility) in 2021
		10	ACTDTY31	0.005	Military Full-time Active Duty - Round 3/1
LightGBM	Accuracy 0.77 Precision 0.67 Recall 0.34 F1 Score 0.45 ROC AUC 0.78	1	TOTSLF21	127.0	Total Amount Paid by Self/Family in 2021
		2	TOTTCH21	96.0	Total Healthcare Charges for 2021 excluding Prescription Medicines
		3	RXSLF21	74.0	Total Amount Paid for Prescription Medicines by Self/Family
		4	OBVSLF21	72.0	Amount Paid for All Office Visits by Self/Family in 2021
		5	TOTEXP21	70.0	Total Healthcare Expenses 2021
		6	OBVTCH21	59.0	Office-Based Provider Visit Charges for 2021
		7	OBVEXP21	47.0	Total Office Based Expenditures in 2021
		8	TOTPRV21	43.0	Total Amount Paid by Private Insurance in 2021
		9	OTHSLF21	39.0	Amount Paid by Self for Other Equipment/Supplies in 2021
		10	POVLEV21	38.0	Family Income as % of Poverty Line Continuous

ROC-AUA, Receiver Operating Characteristic Area Under the Curve; F1, mean score of precision and recall, a score of 1 indicates good performance while low score indicates poor model performance.

## 4 Discussion

Utilizing four Al/ML models, we identified the top determinants for total dental visits in 2021 as out-of-pocket costs, doctor office visits, the use of preventive behaviors in other non-dental care domains (e.g., having glasses/contact lenses), having a diagnosis of attention-deficit/hyperactivity disorder (ADHD), delays in receipt of dental care due to pandemic, emergency visits, and total expenses paid for private insurance. Additionally, our Al models identified years of education and educational degree and level as other top features that predict at least one visit to a dentist (Table 1). When we classified dental visits as either one or two or more than two, we found similarities for top features across this classification than 2 (Tables 1, 2).

For objective 1, XGBoost's performance metrics were superior to all other models. For objective 2, XGBoost and LightGBM performed better than other Al models, but given the binary classification task, LightGBM is considered superior due to its advantage on ROC-AUG. We observed some op features were similar across Al models but not all, with their rankings and importance differing across Al models (see Tables 1, 2). When compared to findings from prior research, our Al models for predicting top features of preventive dental visits and dental visits for more complex care were robust as they identified similar features that have been shown to influence dental visits (6, 11–17, 19, 28–35). For preventive dental care utilization, higher educational level or higher degree (31), age (31), losing teeth, perceived health status (31), and Social Determinants of Health (SDOH) factors (31) were identified as relevant factors by the XGBoost model (36–38). For preventive vs. treatment dental visits that were more than two in 2021, the XBBoost and LightGMB models identified factors that were associated with more severe dental care needs, such as number of emergency visits, having limitations in school, work, or household activities (15, 39), family income (31, 37, 38) or level of household poverty (37, 38), having private insurance, total costs paid by self and or family, total health expenditures, and a person's age (31).

This data report provides several avenues for improving the Al models. First, MEPS data can be pooled across years to increase sample size, before applying transformation, classifications of any sort and before running any Al models. Second, out-of-pocket costs and number of dental visits in a given year can be used to further assess dental needs/severity. Third, the top medical conditions that are associated with those who visit dentists more often can be identified. Fourth, Al models can be developed and trained to determine to what extent having delayed visits to dentists are predictive of having multiple chronic conditions or multimorbidity.

### 4.1 Policy implications

Costs, dental insurance, or socioeconomic barriers make up three of the top ten predictors of any dental visits in 2021, and all top ten predictors of routine vs. therapeutic dental visit patterns. Our findings thus is line with the previous literature that has consistently reported financial and socioeconomic barriers as significant factors for oral health disparities in the U.S. (6, 11–17, 19, 28–33). These results point to household economic precarity and large disparities in wealth and income, in addition to longstanding issues with dental insurance design and integration (30, 33), as potential areas for both long-term and short-term policy interventions to reduce disparities in dental healthcare utilization with the goal to improve oral health outcomes. At least four of the top ten predictors of any dental visits during 2020–2022 were related to disability, mental health, and overall health status (15, 16). These have also been previously linked to barriers for preventative oral healthcare (14–16, 29, 30, 40). For individuals with disabilities, barriers may be due to competing health priorities and resource constraints that affect healthcare utilization (41). Potential policies meant to reduce barriers to care can be designed to reduce process complexity.

### 4.2 Limitations and strengths

We acknowledge several limitations. Given the secondary nature of the MEPS data, our Al models do not account for imbalances originating from the MEPS data collection or measurement, specifically those that exacerbated when data collection were modified during the pandemic of 2020–2022.

MEPS response rates declined during 2020–2021, creating data characteristics that AI models can carry forward or amplify. Nationally representative survey designs impose unique challenges when applying Al and ML approaches and very few software packages provide ways to handle such complex data (42). The Al models were not survey-weighted and did not consider the complex survey design of MEPS incorporating clustering, stratification and survey weighting using primary sampling unit, variance stratum and person weights. As a result, the Al models are skewed and disproportionately reflect those who are more represented in the MEPS and may not be nationally representative. Therefore, our Al models are not generalizable to the U.S. population.

Strengths include our methodological approach grounded in Al/ML techniques and tools helps further provide evidence for social determinants of health and the socioeconomic barriers that have led to the variability of dental care in patient populations during the 2020–2021 public health emergency. This approach also lays groundwork for future strategies to promote oral health access and outcomes for all populations in the U.S.

### 4.3 Future directions

The outlook of utilizing Al/ML approaches to improve the health and wellbeing of populations consistently experiencing greater health challenges remains bright. Our data report describes how the SCHARE platform can be utilized for Al model development to shed light on obstacles and factors that can hinder or facilitate healthcare access, address dental disparities, advance dental and health outcomes research, and to inform policy decisions (e.g., integrating oral and systemic health; so that access to services provided by dentists and/or primary care providers are consolidated and paid for in one office setting). We provide several avenues for future research. Future research can compare differences in features impacting oral health care access and treatment (in 2021) with the most recent MEPS data available (from 2023). *Post-hoc* analysis on the Al models to delineate which of the sub-groups within the identified top features can act as a facilitator or barrier for accessing a dentist, which can be linked to the NIMHD Research Framework. For example, ADHD's role in accessing a dentist may be linked to the individual-level behavioral domain, while societal-level policies may create similar access impediments for people with disabilities. Additionally, future directions include comparing results of unweighted and weighted Al models for national representativeness along with data visualizations to examine systemic variations in how our Al algorithms perform for different population segments included in MEPS.

## Data Availability

Publicly available datasets were analyzed in this study. This data can be found here: https://www.meps.ahrq.gov/mepsweb/data_stats/download_data_files_detail.jsp?cboPufNumber=HC-233.
