# Supplementary material for: Employing AI tools to predict features for dental care use in the United States during the global respiratory illness outbreak
Source: Front Public Health. 2026 Jan 13;13:1692540. doi: 10.3389/fpubh.2025.1692540 (PMC12835335; doi:10.3389/fpubh.2025.1692540)
Supplement: Supplementary file 1 [file Table_1.docx]

**Supplementary Table 1:** Abbreviations and descriptions of variables excluded in our analysis, Medical Expenditure Panel Survey, Year 2021.

| **Variable Name** | **Variable Description** |
| --- | --- |
| DVTTCH21 | Total Dental Care Visit Charges 2021 |
| DVTEXP21 | Total Dental Care Expenditures 2021 |
| OBTOTV21 | Number of Office-Based Provider Visits 2021 |
| DVTSLF21 | Amount Paid by Self/Family for All Dental Care 2021 |
| DVTMCD21 | Amount Paid by Medicaid for All Dental Care 2021 |
| DVTPTR21 | Amount Paid by Private Insurance and Tricare for All Dental Care 2021 |
| DVTMCR21 | Amount Paid by Medicare for All Dental Care 2021 |
| DVTPTR21 | Amount Paid by Private Insurance for all Dental Care 2021 |
| DUID | Panel Number plus Encrypted Dwelling Unit Identifier |
| VARSTR | Variance Estimation Stratum 2021 |
| DUPERSID | Person ID (Dwelling Unit Identifier plus Person Identifier) |
| FAMWT21F | Final Family Weight 201 |
| VARPSU | Variance Estimation Primary Sampling Unit 2021 |
| DOBMM | Date of Birth: Month |
| FAMWT21C | Poverty Adjusted Family Weight Current Population Survey Family on 12/31/2021 |
| PANEL | Panel Number |
| PERTWT21F | Final Person Weight 2021 |
